# Public health financing in Brazil (2019–2022): an analysis of the national health fund and implications for health management

**DOI:** 10.3389/fpubh.2025.1568351

**Published:** 2025-06-09

**Authors:** Luiza Nunes Marinho, Stephen M. Campbell, Isabela Barboza da S. T. Amaral, Rebeca Reis e Silva, Brian Godman, Johanna C. Meyer, Isabella Piassi D. Godói

**Affiliations:** ^1^Institute of Pharmaceutical Sciences, Federal University of Rio de Janeiro, Rio de Janeiro, Brazil; ^2^School of Health Sciences, University of Manchester, Manchester, United Kingdom; ^3^Department of Public Health and Pharmacy Management, School of Pharmacy, Sefako Makgatho Health Sciences University, Pretoria, South Africa; ^4^Institute of Nursing, Federal University of Rio de Janeiro, Rio de Janeiro, Brazil; ^5^Strathclyde Institute of Pharmacy and Biomedical Sciences, University of Strathclyde, Glasgow, United Kingdom; ^6^Center for Neonatal and Paediatric Infection, Institute for Infection and Immunity, City St. George’s, University of London, London, United Kingdom; ^7^South African Vaccination and Immunisation Centre, Sefako Makgatho Health Sciences University, Pretoria, South Africa; ^8^Management Research Group/Center for Health Technology Assessment - Management, Economics, Education and Pharmaceutical Assistance (GEESFAR/NATS/UFRJ) of the Federal University of Rio de Janeiro, Rio de Janeiro, Brazil

**Keywords:** health financing, resource allocation, public health, unified health systems, challenges, health management, Brazil

## Abstract

**Introduction:**

The Unified Health System (SUS) in Brazil provides free, universal health services to all inhabitants of the country. This study aims to describe the allocation of public health resources in Brazil, both overall and across regions, based on National Health Fund (FNS) data from 2019 to 2022. The goal is to provide an understanding of the profile and distribution of resources sourced exclusively from the federal government during this period.

**Methods:**

A quantitative, descriptive study using data extracted from the FNS portal covering the period 2019–2022, along with publications and open data linked to Brazil’s Ministry of Health, was undertaken. Data collection included the resources allocated to health within each of the financing blocks (operational and investment), according to FNS as well as the Transparency Portal of the Office of the Comptroller General for more information about the COVID-19 pandemic.

**Results:**

A total of USD 75.310 billion (406.283 billion BRL) was allocated to health services between 2019 and 2022, with 64.6% allocated to Specialized, Medium, and High Complexity Care and 30.2% to Primary Health Care (USD 21.096 billion). A lower percentage was dedicated to investment actions within the SUS, and there was heterogeneous distribution of resources across the country’s regions, with the Southeast receiving the most resources (38.5%), while the Central-West region received only 7.7%. In addition, more than USD 111 billion (600 billion BRL) was allocated by the federal government to the COVID-19 pandemic response, not exclusively for health-related purposes.

**Conclusion:**

The distribution profile of resources transferred from the FNS reflected population sizes but it is less clear whether resources were allocated based on need. Overall, there was a scarcity of resources allocated to areas such as investment. However, the COVID-19 pandemic represented a considerable impact on government funds. Health and social needs must be assessed and considered going forward to improve the allocation of resources within a unified health system.

## 1 Introduction

The Unified Health System (SUS) represents a significant milestone for Brazil and serves as the primary provider of healthcare for the country’s population (1). Primary Health Care (PHC) services constitute the main gateway for patients to access SUS, offering community-based services focusing on prevention, promotion, and recovery of health (2, 3).

Brazil is currently composed of 26 states and the Federal District, divided into five regions, each characterized by distinct socioeconomic and cultural attributes (4). The country’s Human Development Index (HDI) stands at 0.754 (5), combining indicators of life expectancy, education, and per capita income, which positions Brazil as an upper-middle-income economy with advanced emerging market characteristics (6). Additionally, the country’s Gross Domestic Product (GDP) was approximately 11 trillion BRL in 2023, reflecting continued growth compared to previous years (7).

Brazilian citizens are entitled to public healthcare services through SUS but they may also opt for private health insurance, either individually or via employer-sponsored group plans when available (8). However, from 2019 to 2021, only 22% of Brazilians had private health insurance coverage (9), highlighting the essential role of SUS in providing health services to the majority of the population.

SUS services range from PHC services, including vaccination programs, up to highly complex procedures including organ transplants, covering approximately 90% of healthcare interventions across the country (10). One of the most recognized and widely known initiatives under SUS is the National Immunization Program (Programa Nacional de Imunização, PNI), established in the 1970s. Under this scheme, coupled with public health initiatives, infant mortality (for children up to 5 years old) reduced from 212 deaths per thousand live births in 1940 to just 14 deaths per thousand live births in 2019 (11). Today, more than 40 types of vaccines are available to children, adults, and the older adult as part of Brazil’s National Vaccination Calendar (12). During the COVID-19 pandemic, from January 22, 2020, to March 10, 2023, Brazil distributed over 500 million doses of COVID-19 vaccines at a cost of approximately 38 billion BRL (~USD 7 billion) (13, 14).

Among the laws that regulate SUS, Law No. 8142 of 1990 (15) addresses the context of financing health actions, involving federal, state, and municipal levels (16), as well as the importance of social oversight within SUS. In this regard, efforts have been made to ensure transparency, particularly for the public, concerning the allocation of resources to SUS activities, including through platforms such as the National Health Fund (FNS). The FNS provides accessible information to the public on costing, investments, and financing within SUS via an open-access website (17), aiming to foster social engagement in decision-making. These strategies and tools are essential for promoting informed policy-making, enhancing transparency, and providing accessible information for citizens. Unfortunately, it remains challenging to obtain detailed data on each of the health financing blocks and, particularly, data related to the municipal and state health spheres (18, 19).

It is important to note that the budgetary decision-making process follows several steps, including preparation, approval, execution, and control of the approved budget. According to the Federal Constitution, the federal government budget must be prepared annually by the Executive Branch and authorized by the Legislative Branch, in the form of the Annual Budget Law, which is then monitored by agencies such as the Court of Auditors (20). Until December 2017, the allocation of resources for public health services in Brazil was organized into six financing blocks: (i) PHC; (ii) Medium and High Complexity Outpatient and Hospital Care; (iii) Health Surveillance; (iv) Pharmaceutical Assistance; (v) SUS Management; and (vi) Investments in the Health Services Network. In 2017, these financing blocks were consolidated into two categories under Ordinance 3,992: (i) Costing block, which is responsible for maintaining existing public health actions and services provided by SUS; and the (ii) Investment block, which covers resources allocated for the expansion and improvement of public health services (21–23). It is important to reinforce that before 2019, Primary Health Care (PHC) financing by the SUS followed the Basic Care Floor (PAB) model. This model consisted of a fixed component with automatic *per capita* transfers adjusted by socioeconomic and demographic indicators, and a variable component linked to incentives for strategic actions including family health teams and quality programs and offering budgetary predictability; however, with limited relation to performance targets. With the implementation of new regulations under Ordinance MS No. 2979/2019, with the Previne Brasil Program (24), this model was replaced by four components. These included (i) weighted capitation (based on population size and vulnerability profile), (ii) payment-for-performance linked to quality indicators, (iii) maintenance of incentives for specific programs, and (iv) a per capita transitional incentive. Overall, linking funding to effective coverage and the results achieved by PHC teams.

Between 2019 and 2022, the world faced COVID-19 pandemic. In Brazil, the first confirmed case occurred in March 2020, leading to numerous demands and adjustments in the public health budget. Activities included the creation of new laws (25–27) to support the various health services required during the emergency situation. The pandemic had a severe impact on the Brazilian population, resulting in over 699,000 deaths and approximately 37 million confirmed cases, positioning Brazil as fourth globally in deaths per capita and per 100,000 inhabitants (14).

The pandemic presented numerous challenges and demands for public health services, notably increasing SUS expenditures, particularly in medium-and high-complexity care associated with hospitalizations in Intensive Care Units (ICUs) and specialized treatments. Additionally, SUS financing had to be restructured to meet emerging needs, including the expansion of ICU beds and the acquisition of critical medical equipment (28). While demands for services such as hospitalizations and vaccinations increased, Bigoni et al. (29) demonstrated a reduction in certain other services, especially during the first quarter of the pandemic, including transplants and non-urgent surgeries.

Several studies have examined economic analyses related to healthcare resource allocation by Brazil’s federal government (30–34) as well as by state (19, 35) and municipal governments (18, 36, 37). However, there remains limited understanding regarding the distribution profile of public health resources during the COVID-19 pandemic within SUS, despite the existence of open-access databases such as the FNS and the Public Health Budget Information System (SIOPS) ((38); FNS, 2024). Moreover, the pandemic introduced additional challenges to public health management, including lockdowns and restrictive measures, which affected the care of patients (39–41) and disrupted routine vaccination activities (42–44). These factors highlight the importance of examining and analyzing the economic policies and strategic responses adopted during this period.

Given the scarcity of recent national-level studies demonstrating the distribution and allocation of public health resources, a research project titled *Public Health Financing in Brazil and the Relevance of Public Health Databases: Implications for Health Management* has been underway since 2023. To date, two publications have emerged from this project, both involving descriptive evaluations of resource allocation profiles at the local level, the Macaé municipality (18) and at the state level, the Rio de Janeiro state (19). The present study represents the final stage of this project, focusing on a national-level evaluation of federal management between 2019 and 2022.

Consequently, the aims of this study are to analyze the profile of public health resource distribution based on the FNS database, covering key healthcare sectors, including Primary Health Care (PHC), Medium and High Complexity Care, and Pharmaceutical Assistance within SUS. Focusing on the Costing and Investment financing block, this research examines funding allocation patterns to better understand public health spending during a critical global health crisis. Furthermore, seeking to enhance transparency and understanding of the allocation of federal public health resources and, most importantly, to encourage greater reflection on public health financing in Brazil.

## 2 Methods

### 2.1 Study type and period

This is a quantitative descriptive study aimed at demonstrating the distribution profile of the transfer of public health resources in Brazil between 2019 and 2022, from the perspective of the SUS, sourced from the National Health Fund (FNS). Data was extracted from the National Health Fund platform, including the total amounts of transfers by financing blocks (Costing and Health Investments). Additionally, information on regulatory instruments including the Multi-Year Plan (2020–2023) (45) and the Budget Guidelines Law (2020) (46), obtained from the Ministry of Planning and Budget portal [(20), Access: https://www.gov.br/planejamento/pt-br/assuntos/orcamento]. These sources were consulted to obtain pertinent values for the years 2019–2022. Data selection, collection, and extraction were conducted independently by two researchers under the supervision of the project’s supervisor (IPDG).

### 2.2 Study stages

The FNS platform (Access: https://consultafns.saude.gov.br/#/consolidada) was used to extract the annual and total values related to transfers to the SUS financing blocks, which are Costing and Investment, between 2019 and 2022 in Brazil (FNS, 2024). Each of the SUS financing blocks (Costing and Investment) includes areas related to services and actions including Pharmaceutical Assistance, Specialized Care and Medium-and High-Complexity Care, PHC, Health Surveillance, and SUS Management, consolidated into a single block.

During the data collection and extraction process covering the period from 2019 to 2022, data were obtained from the FNS portal, as well as from documents provided by the federal government that offered greater detail on the allocation of public health resources, particularly during the pandemic (45, 46). The homepage of the FNS platform was accessed, and the option “Consultation and Transfers” was selected, followed by “Consolidated Payment Consultation,” tracking all financial transfers to each of the Brazilian states during each year of analysis. The data extracted from these complementary documents was presented descriptively, whereas data from the FNS portal were included in the Tables and Graphs. It is important to note that in Brazil, the management of public health services involves resources from the federal government, states, and municipalities; however, our data collection focused exclusively on information made available at the federal level. Additionally, an Excel-based form was used to assist in the data extraction process. The data collected did not include resources subsequently allocated at the municipal and state levels, only federal values obtained from the FNS database.

After collecting the data regarding public resources transferred to each financing block for each state, covering the period between 2019 and 2022, the data were compiled into a single database for further analysis. Table 1 shows the sources used for data selection.

**Table 1:** Data selection sources.

Item	Descriptive
Database	FNS*
Period of study	2019–2022
Period of data access	February/March 2024
Transfer of resources (SUS financing blocks)	Costing and Investment
Areas of health actions and services (for each SUS financing block)	Pharmaceutical Services, Specialized Care, Medium and High Complexity, Primary Care, Health Surveillance, and SUS Management
Confrontation of the Health Emergency—COVID-19 (2020 and 2021)	Resource transfers obtained in the investment and costing blocks associated with the COVID-19 pandemic

*FNS, 2024. Accessed on 19 February 2024.

In areas including Specialized Care, Medium and High Complexity Care, and Pharmaceutical Services, certain service types tied to allocated resources were tracked, when available, through the FNS platform. Additionally, it was possible to monitor financial resources directed toward COVID-19 emergency response efforts. These funds were linked to both the Costing and Investment blocks for the years 2020 and 2021.

In particular, the resources involving actions from the Costing block were verified including the promotion of pharmaceutical assistance and strategic supplies in PHC, organization of pharmaceutical assistance in SUS, and financial support for the acquisition and distribution of medicines, from the specialized component of pharmaceutical assistance.

For PHC, collected data included resources applied to the public resources involved in services including Primary Care Floor, Structuring of the PHC Service Network, and Expansion of Basic Health Units (UBS). This reinforces that the PHC allocation was composed of a fixed part, primarily determined by the number of people registered in PHC units at that time, and a variable part related to adherence to certain federal strategies and programs. This was in addition to other components of financial incentives provided by the federal government (3).

### 2.3 Data analysis

The data analyses included the profile and distribution of public resource transfers, originating from the FNS, applied to health nationally, involving the five Regions of Brazil, and covering the general panorama in monetary terms (BRL and USD) of each financing block (Costing and Investment). The costing block is intended to finance actions including consultations and exams in health units, the purchase of medicines for patients, and the payment of health teams. Meanwhile, the investment block is associated with expenditures related to the structuring and expansion of the health service network including an increase in the number of health units (21–23). When available, details involving some areas covering these blocks, including Pharmaceutical Assistance, Specialized Care, Medium and High Complexity, were analyzed. In addition to the general data regarding each of the financing blocks applied in the annual assessment for the period between 2019 and 2022, an analysis was conducted on the distribution of general resources from the FNS across the regions of Brazil. However, the values pertaining to each type of action within the respective financing blocks, by region, were not detailed.

To gather information on the federal resources allocated to various services and actions related to the COVID-19 pandemic, data available in the FNS database for the years 2020 and 2021 were reviewed, with, as mentioned, the first case recorded in 2020. Considering some limitations of public databases, such as missing records and/or lack of data updates, a complementary search was conducted on the Transparency Portal of the Office of the Comptroller General of the Union, which is publicly accessible (47) (Access: https://portaldatransparencia.gov.br/coronavirus). It is worth noting that the federal resources obtained from the Transparency Portal cover various services and demands, and are not exclusive to the health sector. During this period, the federal government financed actions including emergency aid (direct income transfers to informal workers to mitigate the economic effects of the pandemic), credit and support for businesses, and investments in education, including expenses related to remote teaching and the purchase of equipment for public schools.

The Google Sheets^®^ platform and the 2010 version of Microsoft Excel® were utilized to assist with the analyses involving frequency (absolute and relative), stratification, and tabulation of values, as well as the creation of graphs to better illustrate the distribution profile of public resources sourced from the FNS, associated with federal management between 2019 and 2022. Given the short analysis period of only 4 years, it was not feasible to perform a time series analysis (48). It is noteworthy that the focus and analyzes carried out in this study covered the period of a certain government in Brazil, conducted between 2019 and 2022, and not an exclusive assessment of the pandemic in the country.

In addition, we present both the percentage and *per capita* values for each region of Brazil, based on the global resources from the FNS financing blocks. The *per capita* values were calculated using 2022 population data from IBGE (49). For comparison purposes, the conversion rate established by the World Bank for Purchasing Power Parities (PPP) was used (2021, 1 USD = 5.39 BRL) (50). For the calculation of *per capita* values related to the distribution of public resources for each region of the country, whether for general financial resources or specifically for pharmaceutical assistance, the population data from 2022, provided by IBGE, were used. It is important to note that the values presented in USD use commas to separate the thousands and periods before the decimal point. In addition, values presented in BRL use periods to separate the thousands and commas to separate the decimal places. We believe in the importance of demonstrating data in both BRL and USD.

## 3 Results

### 3.1 Financing of public health services in Brazil (2019–2022)

Based on the data from the FNS for the period 2019 to 2022, the amount of 406.283 billion BRL (USD 75.377 billion) was allocated to SUS. Of this, 398.833 billion BRL (USD 73.995 billion) was allocated for the Costing block and 7.450 billion BRL (USD 1.382 billion) for the Investment block. Table 2 shows that overall allocation of funding for both the Costing and Investment blocks increased during 2020 and 2021 compared with 2019, and then fell in 2022. Most of the public funding was allocated to SUS Public Health Actions and Services (98.2%).

**Table 2:** Transfer of FNS resources for SUS financing to the costing and investment blocks between 2019 and 2022.

Year	Costing (USD)	%	Investment (USD)	%	Total (USD)
2019	15400618199.26	98.1	294245677.92	1.9	15694863877.18
2020	20822966948.42	97.4	548177844.90	2.6	21371144793.32
2021	19267277418.55	98.5	285858983.30	1.5	19553136401.85
2022	18504235720.96	98.6	253917150.46	1.4	18758152871.42
Total	73995098287.19	98.1	1382199656.58	1.9%	75377297943.77

FNS, 2024. Accessed on 19 February 2024.

Based on FNS data, in the period 2019 to 2022, of the 406.283 billion BRL (US$ 75.377 billion) that were invested considering the Costing and Investment blocks, the North region was allocated 8.5%, the Northeast 30.5%, the Midwest 7.7%, the Southeast 38.5% and the South region 15%. Among the resources transferred, the Southeast region was the one that received the largest amount in terms of resources, with approximately 156 billion BRL (USD 28.993 billion) while on the other hand, the Central-West region received the smallest share of these resources, amounting to approximately 31 billion BRL (USD 5.832 billion) (see Table 3). Additionally, the *per capita* values allocated to health from the FNS were calculated for each region of Brazil, considering the average value between 2019 and 2022: 1.979 BRL (USD 367.23) for the North region, 2.262 BRL (USD 419.74) for the Northeast region, 1.928 BRL (USD 357.73) for the Central West region, 1.840 BRL (USD 341.44) for the Southeast region, and 2.028 BRL (USD 376.29) for the South region.

**Table 3:** Transfer of health resources from the FNS to each region of Brazil from 2019 to 2022.

Region	Value (USD)	%	Number of inhabitants	*Per capita* (USD)
North	6373395268.94	8.5	17,354,884	367.23
Northeast	22942775356.71	30.5	54,658,515	419.74
West Center	5827264079.19	7.7	16,289,538	357.73
Southeast	28968378299.30	38.5	84,840,113	341.44
South	11265484940.39	15.0	29,937,706	376.29
Total (USD)	75377297944.53	100	203,080,756	371.16

The public resources presented are associated with the funding and investment blocks of each region in the period between 2019 and 2022. Number of inhabitants of 2022, according to IBGE. FNS, 2024. Accessed on 24 November 2024; IBGE. Accessed on 09 December 2024.

**Figure 1:**
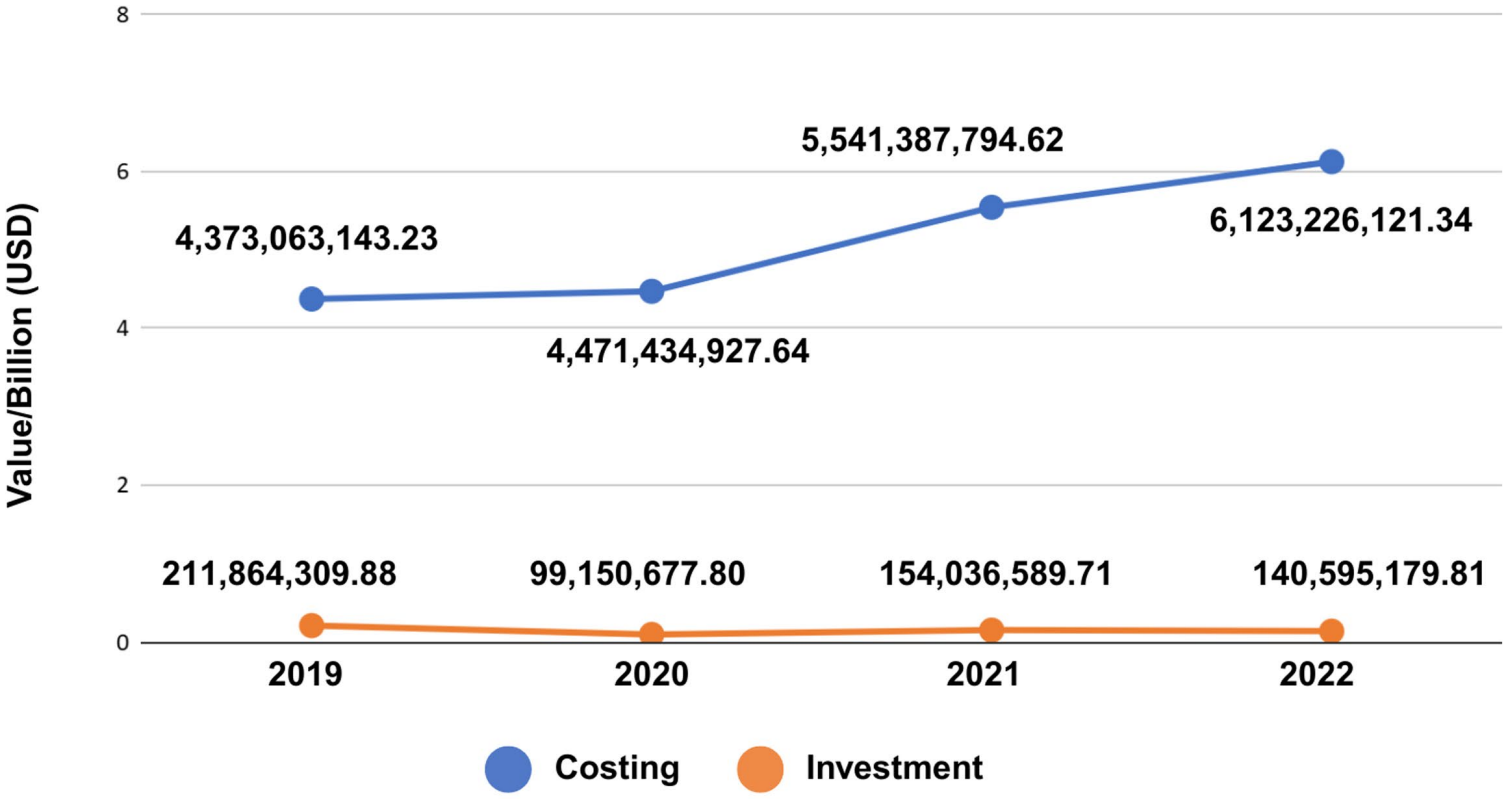
Transfer of public resources destined to PHC (2019–2022) in Brazil, according to FNS.

Within the SUS Public Health Actions and Services, during the period 2019 to 2022, a total of 50.401 billion BRL (USD 9.342 billion) was allocated for PHC services and actions carried out within basic health units and family health strategies (PHC units) (85.262 billion BRL; 75%), structuring of the service network (2.952 billion BRL; 3%), and construction and expansion of basic health units (86.918 million BRL; 0.08%). As shown in Figure 1, 110.544 billion BRL (USD 20.509 billion) and 3.264 billion BRL (USD 605.646 million), respectively, were allocated from the FNS to the Costing and Investment blocks for PHC. Figure 1 shows the overview of the resources allocated to PHC, coming from the FNS for the period from 2019 to 2022, considering the Costing and Investment blocks. Our results demonstrate a growing trend in health financing in Brazil, based on data from the federal government. Additionally, we highlight a greater increase in resources allocated annually to Specialized, Medium and High complexity care, in the period between 2019 and 2022.

**Figure 2:**
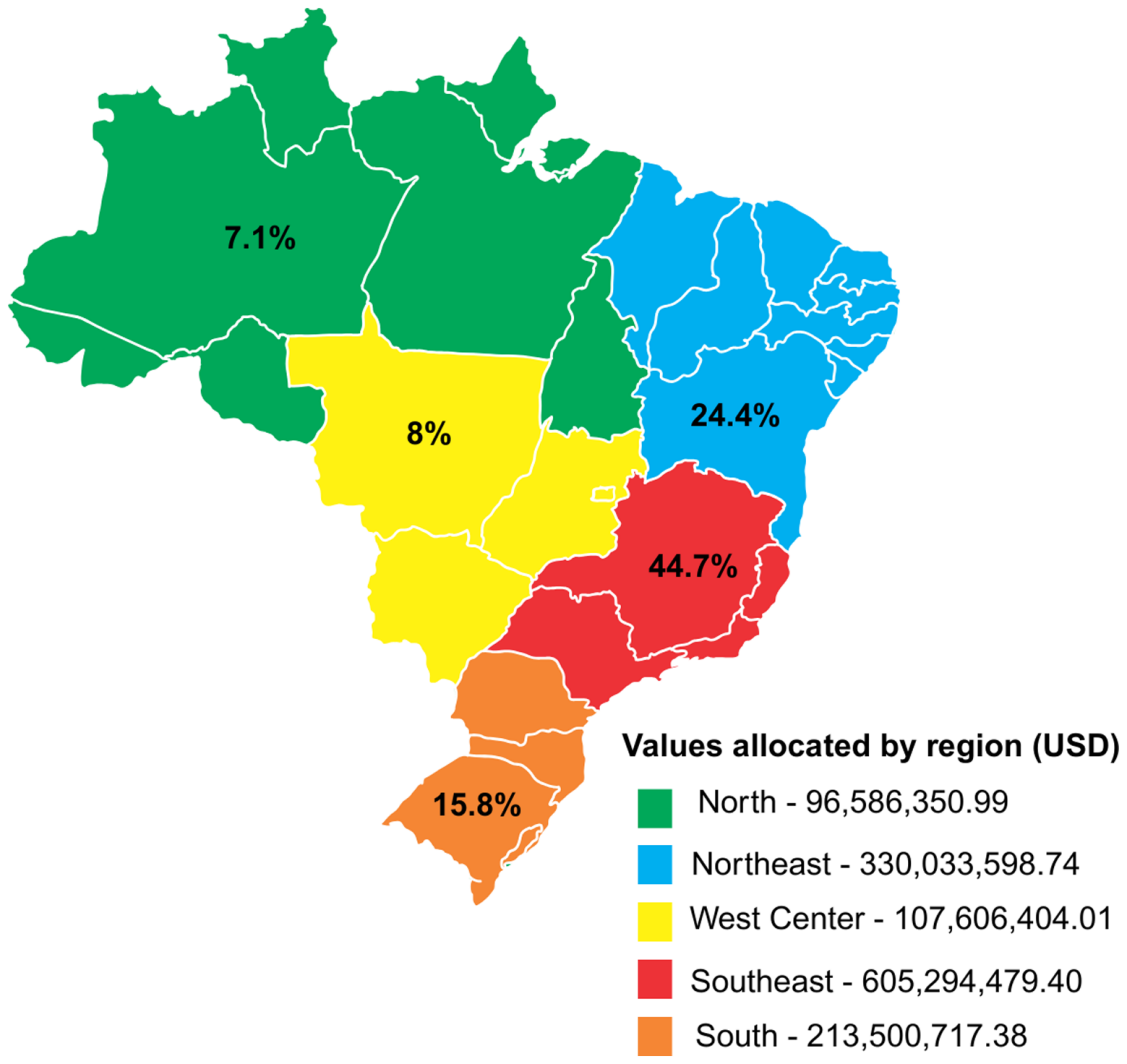
Transfer of public resources destined to Pharmaceutical Assistance by region of Brazil between 2019 and 2022, according to FNS.

Figure 2 shows the proportional distribution of the amounts transferred from the FNS to the services and actions of Pharmaceutical Assistance among the five regions of Brazil. There is a distinction in the profile of resource transfer, especially between the North region (USD 96.586 million; 7.1%), when compared to other regions of the country such as the Northeast (USD 330.033 million; 24.4%), South (USD 213.500 million; 15.8%), Southeast (USD 605.294 million; 44.7%) and Central-West (USD 107.606 million; 8.0%). We have seen that the monetary distribution of public resources allocated to Pharmaceutical Assistance of each region of the country is aligned to the population size distribution of each region, respectively, as presented in Table 3. However, this does not appear to take into account key issues such as need as opposed to population numbers. In addition, the *per capita* values allocated to pharmaceutical assistance services were calculated for each region, considering the average between 2019 and 2022 for this health sector: 30.02 BRL (USD 5.57) for the North region, 32.55 BRL (USD 6.04) for the Northeast region, 35.63 BRL (USD 6.61) for the Central-West region, 38.43 BRL (USD 7.13) for the Southeast region, and 38.43 BRL (USD 7.13) for the South region.

### 3.2 Distribution and allocation profile of public resources in Brazil associated with the costing of health actions (2019–2022)

Table 4 shows the amounts allocated by the FNS to the financing areas applied to the Costing block during the period of this study. In addition, 41.142 million BRL (USD 7.626 million), which corresponds to 0.01% of the total amount allocated to the costing block, were directed to SUS management actions, according to the FNS data. We highlight that Specialized, Medium and High complexity care represented the majority of resources allocated to health, according to the FNS database, in relation to the total percentage, as well as the annual increase for the study period.

**Table 4:** Financing of Health Actions related to the Costing Block, from the National Health Fund (FNS), between 2019 and 2022 in Brazil.

Year	Pharmaceutical assistance (USD)	Specialized care, medium and high complexity (USD)	Primary care (USD)	Health surveillance (USD)	Total (USD)
2019	334077368.27 (2.2%)	10192168746.01 (66.2%)	4373063143.23 (28.4%)	501011167.53 (3.2%)	15400320425.04
2020	329735784.97 (2.2%)	9785530117.44 (64.4%)	4471434927.64 (29.6%)	504942390.91 (3.8%)	15091643220.96
2021	310088248.79 (1.6%)	12562467219.11 (66.3%)	5541387794.62 (29.2%)	543139781.08 (2.9%)	18957083043.60
2022	368355967.90 (2.0%)	11354826229.68 (61.5%)	6123226121.33 (33.1%)	653536127.46 (3.4%)	18499944446.37
Total (USD)	1342237369.90	43894992312.24	20509111986.82	2202629466.98	67948991135.97

FNS, 2024. Accessed on 19 February 2024.

Within the scope of Pharmaceutical Assistance, a total of 7.234 billion BRL (USD 1.341 billion) was allocated, including 5.180 billion BRL (USD 960.2 million) for the promotion of pharmaceutical assistance, 224.880 million BRL (USD 41.684 million) for the organization of pharmaceutical assistance, and 335.483 million BRL (USD 61.826 million) for the acquisition and distribution of medicines under the Specialized Component of Pharmaceutical Assistance.

After analyzing the data collected, from the FNS referring to the records associated with Specialized Care, Medium and High Complexity, involving the Costing block, it was found that 236.594 billion BRL (USD 43.894 billion) were allocated to this area, which represented 64.6% of the resources forwarded during the period 2019 to 2022 as shown in Table 4. Among the actions and services, the following stand out. Firstly, support for the Maintenance of Health Units, with 367.915 million BRL (USD 68.198 million) and Population Health Care in High Complexity Procedures, with 211.273 billion BRL (USD 39.162 billion). In addition, 52.320 million BRL (USD 9.698 million) were allocated to the Operationalization of the National Transplant System (SNT), and 7.472 billion BRL (USD 1.385 billion) were allocated to the Confrontation of the Health Emergency registered in 2021 and 2022 associated to the pandemic.

In the context of the COVID-19 pandemic, it was found that 32.547 billion BRL (USD 6.033 billion) were used to promote health services based on data available for the years 2020 and 2021, according to the FNS database. Specific details about the various health actions associated with these resources are not provided. In addition, according to the Transparency Portal of the Office of the Comptroller General of the Union, this revealed that approximately 524.02 billion BRL (USD 97.22 billion), 116.20 billion BRL (USD 21.56 billion), and 11.10 billion BRL (USD 2.06 billion) in federal resources were allocated to combating the pandemic, totaling 651.32 billion BRL (USD 120.84 billion), associated only with this federal government source (47). The significant amount obtained from the Transparency Portal covers various actions, many of which are not related to health and include the payment of emergency aid (direct income transfers to informal workers).

### 3.3 Distribution and allocation profile of public resources in Brazil associated with the investment block of health actions (2019–2022)

The Investment block represented only 7.450 billion BRL (USD 1.382 billion), or 1.9% of the resources allocated to health coming from the FNS during the period 2019 to 2022. In this context, it is observed that PHC (53.9%) and Specialized Care, Medium and High Complexity (43.5%) were the areas in which most financial resources were allocated. Contrary to that observed in the resource transfers allocated to the Costing block, most of the amounts applied to investment actions were directed to PHC.

## 4 Discussion

Health financing in Brazil involves multiple actors and sources of funds through a combination of public and private resources, including taxes, social contributions, fees, and transfers from the federal government, states, and municipalities. This study shows that among the resources allocated to SUS public health services (BRL 406.283 billion / USD 75.377 billion) through the FNS (National Health Fund), most are allocated to the Costing block, which accounts for BRL 398.833 billion / USD 73.995 billion (98.0%), compared to BRL 7.450 billion/USD 1.382 billion (1.8%) allocated to the Investment block. This distribution profile of federal public resources demonstrated in our study confirms a modest percentage of resources allocated to areas such as investment in the health network. Viana et al. (19), in an evaluation of the allocation of federal public resources for the state of Rio de Janeiro, also revealed that between 2015 and 2018, only 1.68% of the funds were allocated to investments (19). A similar finding was observed in a study conducted by Silos et al. (18), which evaluated FNS transfers to the municipality of Macaé, noting that only 2.95% of the funds were allocated to this area. These results may highlight the importance and responsibility, particularly of local administrations, in promoting efforts to expand and improve the health service network available to their respective populations.

According to the report presented to the Brazilian Senate to account for the resources allocated in the country during the COVID-19 pandemic, investments in health were recorded not only temporarily but also beyond this period of health crisis in the country, associated with the structuring of specialized healthcare units, education, and health training. The main highlights were the restructuring of university hospitals and the transfer of approximately R$ 248 million (51). Additionally, it is worth noting that for some months, the Brazilian government made Emergency Aid available (April/2020 to October/2021) to thousands of Brazilians, which amounted to approximately 359 billion BRL, considering the worsening socioeconomic conditions of many families in the country such as unemployment during the pandemic (52). In this context, based on the data from our study, more than BRL 600 billion were allocated to the COVID-19 pandemic response between 2020 and 2022 (47). However, the majority of these resources were not directed toward the promotion of health services.

Our study demonstrated that considerable resources are now allocated to health in Brazil at the federal level. Our study also highlights that health demands tend to increase and are subject to unforeseen situations, such as those experienced during the COVID-19 pandemic. This trend is evident in Table 4 and Figure 2, which show the growing public health resources, especially those applied to the Costing block, between 2019 and 2022 in the country. However, investments in infrastructure improvement, including for hospitals, were scarce during this period. Consequently, we believe that the priority given to public health in 2020 and 2021 was temporary. Constitutional Amendment 95 of 2016 (53) was not altered, and the federal resources approved to address the pandemic were considered extraordinary credits, part of the so-called “War Budget” (54), which was not accounted for within the spending ceiling. This War Budget increased the contribution of federal resources during the health crisis resulting from COVID-19, as well as the relaxation of fiscal, administrative and financial rules during this period (54). It is worth mentioning that since 2017, federal resources for financing health in Brazil have been limited by EC95 in recent years (36). Consequently, there has been a reduction in the Union’s participation in the financing of public health actions and services, and in contrast, a progressive increase in financing from the state and municipal spheres. The Institute of Applied Economic Research pointed out that in 2012, the Union’s participation was 45.3%; that of the states and Federal District was 25.3%; and that of municipalities of 29.4%, and already in 2022, the registered participation percentages were 37.6, 28.4, and 34.0%, respectively (22).

The guarantee of universality and comprehensiveness of the SUS is conditioned by overcoming unrealistic budgetary restrictions (55), such as those imposed by Constitutional Amendment 95, which conflicts with the actual health needs of the Brazilian population. Although the distribution of public health resources from the FNS across Brazil’s regions has followed, to some extent, the demographic proportion (as shown in Table 3), it is important to note that the North and Northeast regions, for example, have faced significant socioeconomic challenges such as higher levels of unemployment and a lack of basic sanitation in some municipalities (4), leading to increased health demands, which are not seen in the other regions, such as the Southeast (56). Unfortunately, this situation persists today, underscoring the importance of continuously monitoring the population’s needs to ensure that resource allocation meets the specific demands of these regions (57, 58).

As shown in Table 3 and Figure 2, population sizes appear to have been the principal criteria for resource allocation by the federal government. However, it remains unclear whether the real needs of the population are adequately considered in the distribution of health resources to different states and regions (59) with, as mentioned, considerable economic challenges in some states and regions. Unfortunately, user dissatisfaction due to infrastructure deficiencies and/or access difficulties to services such as surgeries, e.g., long waiting times, is commonly observed in some studies (8, 60), which needs to be addressed going forward. This though requires changes in the resource allocation formula by the Federal government. A study by Rocha et al. (58) shows that, on average, the North and Northeast health regions have significantly fewer ICU beds per resident (4.4 and 4.3 per 100,000 residents) compared to the Southeast region (9.6 per 100,000 residents). Additionally, between 2019 and 2022, the Northeast region experienced the greatest income inequality in the country (61). In terms of general mortality, data from 2022 revealed that the Southeast region had the highest rate at 8.19%, followed by the South at 8.17%, and the Northeast in third place at 7.37%, with factors such as population density and violence being major contributors (62). Moreover, there is a high incidence of infectious diseases in the North and Northeast regions, associated with lower coverage of basic sanitation compared to other regions such as the Southeast (63). These different points need to be taken into consideration in the future.

Encouragingly, after the publication of Ordinance MS No. 2979/2019, resources allocated to Primary Health Care (PHC) were no longer distributed solely based on the number of inhabitants, but began to incorporate a weighted allocation model. This model assigns greater weight to records of individuals in contexts of greater socioeconomic vulnerability, to specific age groups (children and the older adult), and to rural–urban classifications, including additional transfers conditional on the achievement of PHC quality indicators. As a result, it was observed that regions with smaller populations but higher vulnerability rates and good PHC coverage could receive more resources per capita compared to more populated and less vulnerable areas and/or areas with insufficient coverage. Considering the recent changes in the financing model applied to this strategic area of health care, we observe that there are many controversies and differing perspectives regarding critical evaluations of the implemented models (33, 59). In this context, our funding analysis shows that Specialized, Medium, and High Complexity Care received BRL 236.594 billion (USD 43.894 billion), while Primary Health Care (PHC) received BRL 110.544 billion (USD 20.509 billion), further highlighting a continued significant disparity in resource allocation. Studies by Stange et al. (64) and Brundtland (65) suggest that this disparity reflects the prioritization of more complex and higher-cost treatments, including hospital admissions and specialized procedures, at the expense of PHC services. Consequently, while PHC is the foundation of the health system and essential for health promotion and disease prevention, it often receives fewer resources, which can undermine its effectiveness and, consequently, increase the demand for more complex services, as shown in Table 4. These findings align with the results of other studies addressing public health financing from the perspective of the SUS in Brazil, particularly in relation to transfers from the federal government (18, 19). This also needs addressing going forward.

Between 2019 and 2022, significant funding was allocated to Specialized, Medium, and High Complexity Care, which accounted for 59.3% of the total resources allocated to health. Areas such as PHC (27.7%), Health Surveillance (2.9%), and Pharmaceutical Assistance (1.8%) received lower proportional funding compared to resources allocated to more complex care, according to the FNS database. Other studies have also shown that PHC services have historically received significantly lower financial resources compared to higher complexity services (65, 66). Additionally, Tekola et al. (67) argued that investing in Health Surveillance is crucial to detect, prevent, and respond to public health emergencies. However, this area received the lowest funding, especially in 2021 (2.9%) as shown in Table 4. As demonstrated by our results, the Southeast (38.43 BRL/USD 7.13) and South (38.43 BRL/USD 7.13) regions receive higher amounts, *per capita*, allocated to the financing of Pharmaceutical Assistance, which may be associated with the greater availability of infrastructure for pharmaceutical assistance. An analysis by geopolitical regions revealed significant inequalities in the adequacy profiles of Pharmaceutical Assistance in Brazil, with the South and Southeast regions showing the best performances (68). This again needs re-evaluation within a universal healthcare service.

Encouragingly during this period, Brazil made significant investments in vaccination services, with the Ministry of Health implementing different federal healthcare financing rules over the last few decades. Constitutional Amendment No. 95/2016 (53), which set a limit of 15% of the 2017 Current Net Revenue for the period 2018 to 2036, had several implications, including the imposition of the “Expenditure Ceiling” (69, 70). This though revealed the lack of “practical sustainability” of the “Expenditure Ceiling” in the health sector, given the dynamic and growing demands of this universal system, as demonstrated in Table 4.

We acknowledge there are a number of limitations in this study. These include difficulties in extracting certain data and the lack of previous studies covering the same period, which hindered comparisons with other national studies at a similar level of detail. Considering that the SUS is financed by all three levels of government, it is also important to note the limitation of using only data from the National Health Fund, which reflects only the federal government’s contribution. This approach restricts a more comprehensive view of the system’s overall financing. Furthermore, we were unable to access data on resource transfers involving state and municipal management during the study period, which limits the possibility of a more comprehensive discussion regarding health developments and outcomes during the pandemic, considering the active participation of the three levels of government. Additionally, it was not possible to present descriptive and quantitative information for each area and their respective activities (e.g., how, when, and with what resources) related to each Financing Block, due to the unavailability of such details on the FNS Portal and some inherent limitations of using public health databases including the underreporting of records. Moreover, given the study period of only 4 years (2019–2022), it was not possible to conduct a time series statistical analysis. Nevertheless, a descriptive trend indicating an increase in the allocation of public health resources was observed in 2020 and 2021, likely associated with the numerous public health demands generated by the COVID-19 pandemic.

## 5 Conclusion

From this descriptive study, it was found that between 2019 and 2022, approximately BRL 406.283 billion (USD 75,377 billion) were allocated to health, involving the Costing block (BRL 398.833 billion / USD 73,995 billion) and the Investment block (BRL 7.450 billion/USD 1,382 billion), coming from the FNS. Public resources from the federal government primarily come from the collection of various types of taxes and duties. Our analysis showed that throughout the evaluated period, there was a scarcity of resources allocated to the Investment block affecting any expansion of the network of health units to facilitate increased access as well as improving the infrastructure of public hospitals in the country. Since the period, 2019 to 2022, included the severe public health crisis of the COVID-19 pandemic, the distribution profile, mostly directed to the Costing block, with the main focus on service provision, aligns with expected trends. However, new studies are encouraged to foster greater debates and analysis of the distribution profile of public health resources in the country. It is also essential to resume and expand investments in the post-pandemic period.

The size of the population alongside the demands and needs of the population must be considered in the resource allocation process especially involving the federal government. This study shows that resource allocation is distributed in alignment with population sizes within regions. However, there are socioeconomic weaknesses in regions such as the North and Northeast, including a lack of satisfactory basic sanitation in municipalities directly impacting morbidity and mortality from infectious diseases. While population size may be seen as a proxy for health need, it is less clear whether resources are allocated according to actual health needs within regions and this must be considered in resource allocation rather than just population sizes. Efforts should also be made to “compensate” for investments that were not made in the past, which have implications for health today and certainly for the future. There are many challenges to consolidating a system that promotes and achieves universality and comprehensiveness of health actions for more than 200 million inhabitants, and we will continue to monitor the situation to help provide future guidance.

## Data Availability

The original contributions presented in the study are included in the article/supplementary material, further inquiries can be directed to the corresponding authors.
